# Long-term Response to Vismodegib in a Patient with Gorlin-Goltz Syndrome: A Case Report and Review of Pathological Mechanisms Involved

**DOI:** 10.7759/cureus.5383

**Published:** 2019-08-13

**Authors:** Meghana Kesireddy, Vincent L Mendiola, Bagi Jana, Shrestha Patel

**Affiliations:** 1 Internal Medicine, The University of Texas Medical Branch, Galveston, USA; 2 Hematology Oncology, The University of Texas Medical Branch, Galveston, USA; 3 Oncology, The University of Texas Medical Branch, Galveston, USA

**Keywords:** gorlin goltz syndrome, gorlin syndrome, vismodegib

## Abstract

Nevoid basal cell carcinoma syndrome (NBCCS), also known as Gorlin-Goltz syndrome or Gorlin syndrome, is a rare multisystem disorder with an estimated prevalence of around 1 in 100,000 on average. Vismodegib, an oral smoothened (SMO) inhibitor that blocks the activation of the sonic hedgehog (SHH) pathway, is used in patients with NBCCS. We present an interesting case of a 38-year-old female with Gorlin-Goltz syndrome and her response to vismodegib therapy over two and a half years. She had an excellent initial response to vismodegib for a year during which all her skin basal cell carcinoma (BCC) lesions decreased in size considerably; her dentigerous cysts remained the same size. Though she continued therapy despite several side effects, this was only followed by tumor regrowth and the emergence of new BCC lesions in a more aggressive manner. We discussed the proposed mechanism of resistance to vismodegib (based on our case and literature review) along with its clinical implications. Our case highlights that vismodegib resistance might lead to progression of Gorlin syndrome to a more aggressive version, and points out the need to determine the optimal regimen (combining vismodegib with other agents) and optimal therapy duration (continuous treatment vs. discontinuation after best response) for treatment of NBCCS.

## Introduction

Nevoid basal cell carcinoma syndrome (NBCCS) also known as Gorlin-Goltz syndrome or Gorlin syndrome is a rare, autosomal dominant, multisystem disorder with an estimated prevalence of around 1 in 100,000 on average [1-2]. It is caused by germline mutation in the human homolog Drosophila patched (PTCH1) gene [3] or the suppressor of fused (SUFU) gene [3-4], components of the sonic hedgehog (HH) signaling pathway. The overactivation of the sonic hedgehog (HH) pathway is implicated in the development of basal cell carcinoma (BCC) and other tumors in this syndrome [5].

NBCCS is diagnosed clinically with two major criteria (or) one major and two minor criteria [6]. Major criteria include multiple basal cell carcinomas (BCC), odontogenic keratocysts of the jaw, palmar/plantar pits, falx cerebri calcifications, rib deformities, and first degree relative with NBCCS. Minor criteria include medulloblastomas, ovarian fibroma, congenital malformations like cleft lip/palate and other radiological abnormalities. Genetic testing is needed if the clinical diagnosis is uncertain.

Vismodegib, an oral smoothened (SMO) inhibitor that blocks the activation of the sonic hedgehog (SHH) pathway, approved for the treatment of advanced and metastatic BCC, has been used in patients with NBCCS. In a randomized trial, vismodegib has been shown to decrease the tumor burden of BCC in patients with NBCCS, and nearly 54% of patients experienced severe side effects like muscle cramps, hair loss, ageusia and weight loss leading to treatment discontinuation [7]. Due to the significant side effects, long term vismodegib studies have not been conducted to determine the potential therapeutic role of vismodegib in NBCCS.

We present an interesting case of a 38-year-old female with Gorlin-Goltz syndrome and her long-term response to vismodegib therapy. She was able to tolerate vismodegib therapy despite its side effects, had an excellent initial response to the drug for about a year followed by tumor regrowth. We also discussed the proposed mechanism of resistance to vismodegib and its potential clinical implications (based on our case and literature review).

## Case presentation

**Figure 1:**
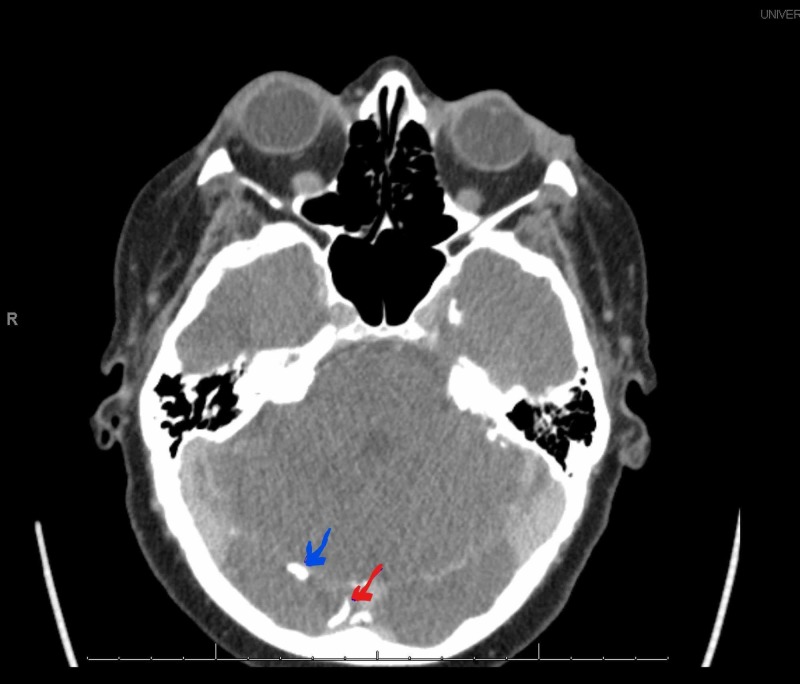
CT head showing calcifications along the falx and tentorium cerebelli. The blue arrow is showing calcifications along the tentorium cerebelli, and the red arrow is showing calcifications along the falx cerebelli.

**Figure 2:**
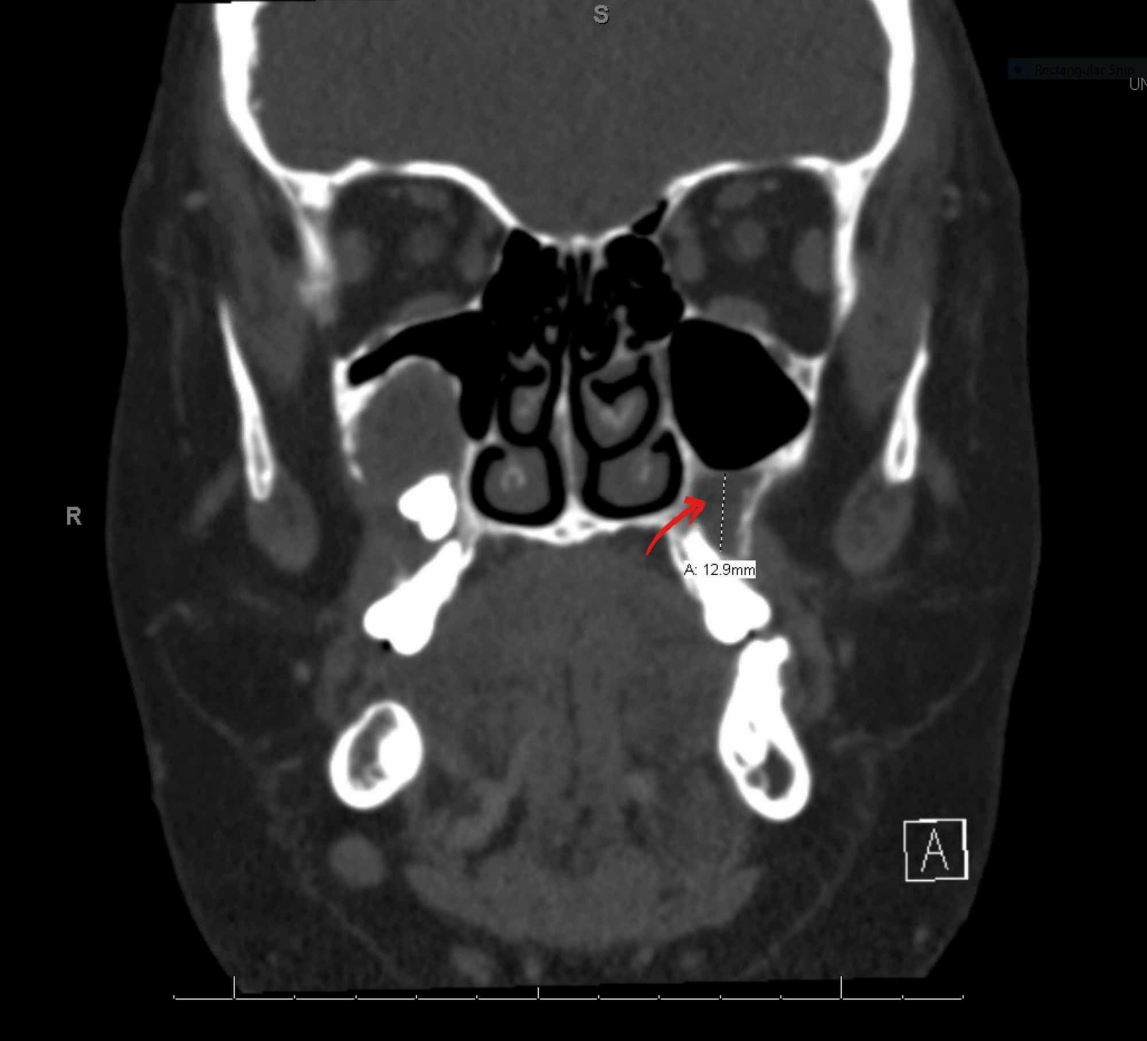
CT maxillofacial and neck showing left maxillary molar (unilocular) dentigerous cyst measuring 1.2 cm x 1 cm x 1 cm. The red arrow is indicating the left maxillary dentigerous cyst.

**Figure 3:**
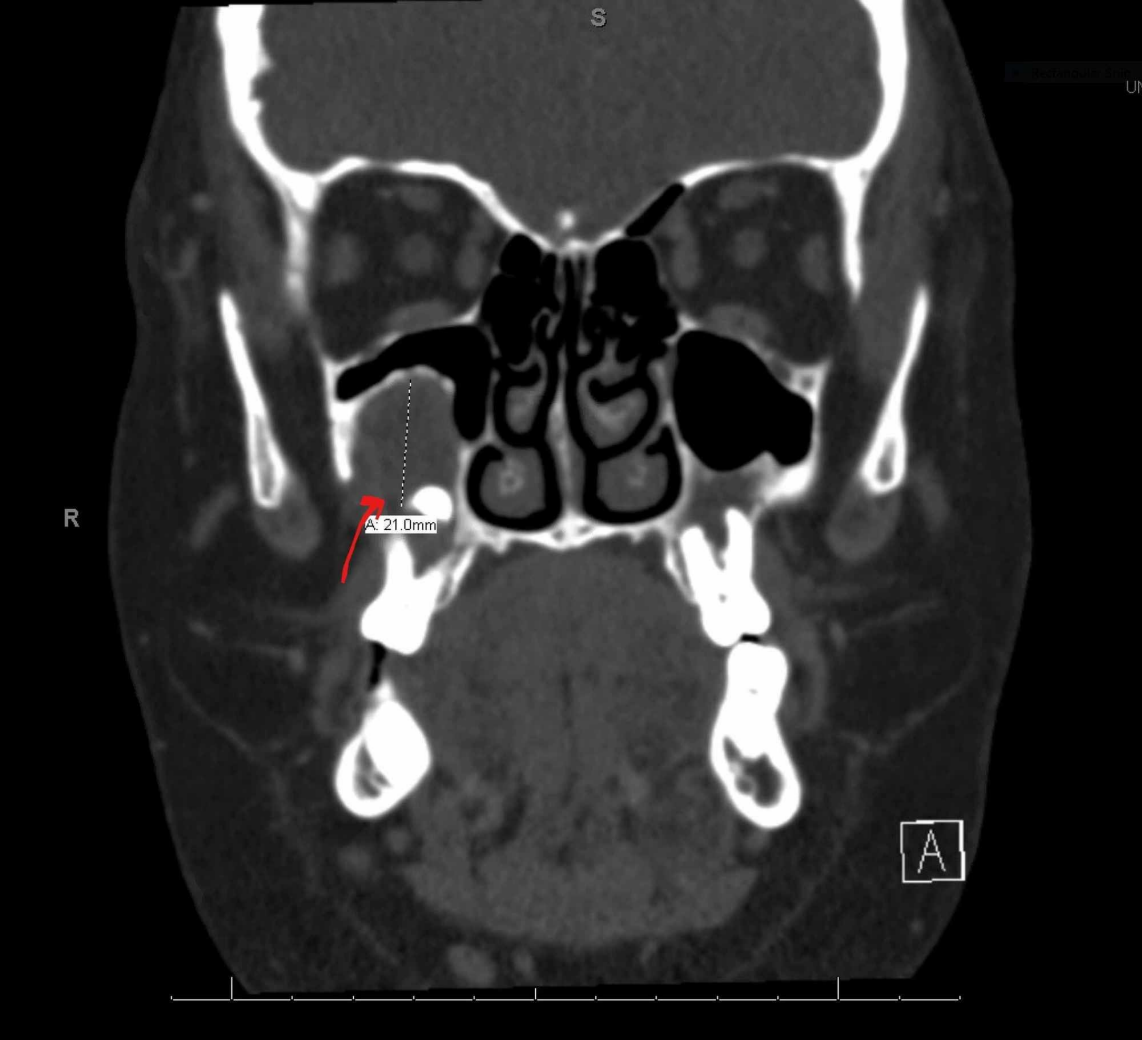
CT maxillofacial and neck showing right maxillary molar (unilocular) dentigerous cyst measuring 2.1 cm x 1.9 cm x 1.6 cm. The red arrow is indicating the right maxillary dentigerous cyst.

A 38-year-old female with a past medical history of spina bifida, jaw cyst excision at the age of 8, and multiple BCC treated with either excision or electrodesiccation and curettage (ED & C) at the age of 25 presented with multiple skin lesions on the scalp, face and the neck. Biopsy of three of those (most prominent) lesions revealed BCC and was treated with ED&C. Further diagnostic workup included CT head which showed calcifications of tentorium and falx cerebelli with bridging of sella turcica; CT maxillofacial & neck which showed two posterior maxillary (one on the right, one on the left, at the level of second-third maxillary molars) unilocular cysts consistent with odontogenic keratocysts; chest X-ray which showed no rib abnormalities, and a pelvic ultrasound which showed no ovarian fibromas (Figures 1-3).

**Figure 4:**
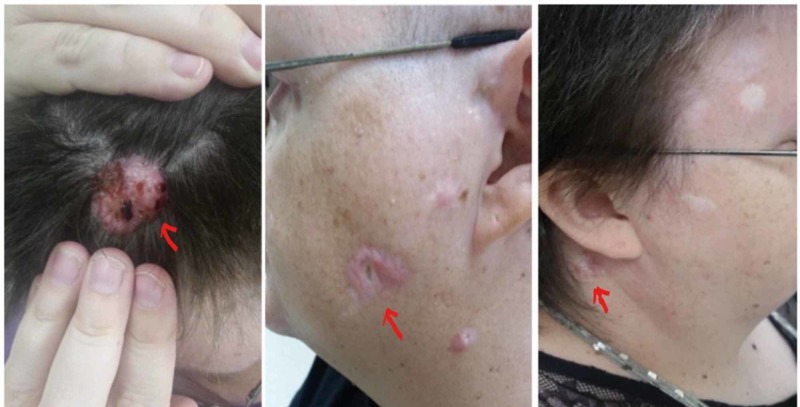
Basal cell carcinoma lesions (shown by arrows) before treatment with vismodegib.

**Figure 5:**
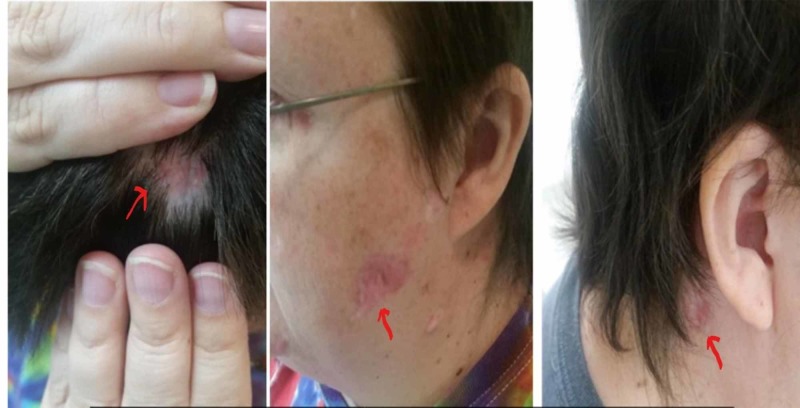
Basal cell carcinoma lesions (shown by arrows) decreased in size significantly after treatment with vismodegib.

Gorlin syndrome was diagnosed using diagnostic criteria [6]. She had two major criteria - multiple BCC, odontogenic keratocysts of the jaw - and one minor criterion - radiologic abnormalities like bridging of sella turcica. Genetic testing (not required for diagnosis in this case) revealed the pathogenic inactivating mutation in PTCH1 gene. Vismodegib, an oral selective SMO inhibitor, 150 mg daily, was started. After vismodegib initiation, for the next 11 months, she did not develop any new skin lesions, all her existing skin lesions decreased in size considerably, and there was no change in the size of her odontogenic keratocysts (Figures 4-5).

Eleven months after vismodegib initiation, she started having an increase in the size of the existing lesions along with new skin lesions in the following pattern. Over the following six months, she had three new BCC lesions - one each on the right temple, the right shoulder, and the left eyelid which continued to grow extensively into the supraorbital ridge and lateral rectus muscle despite debulking. Over the subsequent next nine months, she had 11 new BCC lesions - two BCCs on the left shoulder, one on the right cheek, two BCCs on the right lateral eyebrow/right temporal area, four BCCs on the upper back, lower back, right temple, right temporal hairline, one on left jaw and one on right forehead. Over the next three months, she developed seven new BCC lesions on the right frontal scalp, left frontal scalp, right superior temple, right lateral eyebrow, left neck in the postauricular area. Some of her facial lesions were treated with Mohs surgery, and all the remaining lesions were treated with ED&C.

Throughout these two and half years of vismodegib therapy, she had nausea, dysgeusia (altered taste sensation), grade I diarrhea, bilateral lower extremity cramps, hair loss, amenorrhea as side effects which were not severe enough to warrant discontinuation of therapy. Finally, after 2.5 years of vismodegib therapy, vismodegib was discontinued due to unclear benefit, recurrence of BCC which occurred with an increased frequency as time progressed, and persistent side effects which did not improve with time.

## Discussion

This is an interesting case of Gorlin-Goltz syndrome where i) the skin lesions (basal cell carcinoma) had an excellent and durable response to vismodegib, for almost a year, followed by regrowth of old lesions and the emergence of new lesions implying the development of resistance to vismodegib, ii) the new BCC lesions developed with increased frequency as time progressed despite continued therapy suggesting that the mutations/mechanisms causing vismodegib resistance might be responsible for the development of aggressive version of Gorlin syndrome, iii) the bilateral keratocysts/dentigerous cysts showed no response to the drug implying vismodegib is not an optimal treatment option for keratocysts, and iv) she had many side effects (the leading cause of drug discontinuation in most cases) from vismodegib but were not distressing enough to warrant discontinuation and the side effects did not improve with time.

Below is a brief review of the pathogenesis of NBCCS to understand the proposed mechanisms for the development of vismodegib resistance and its clinical implications. As discussed in the introduction part, overactivation of the sonic hedgehog (HH) signaling pathway is responsible for the development of BCC and other tumors [5].

Normal hedgehog pathway (after embryonic development)

**Figure 6:**
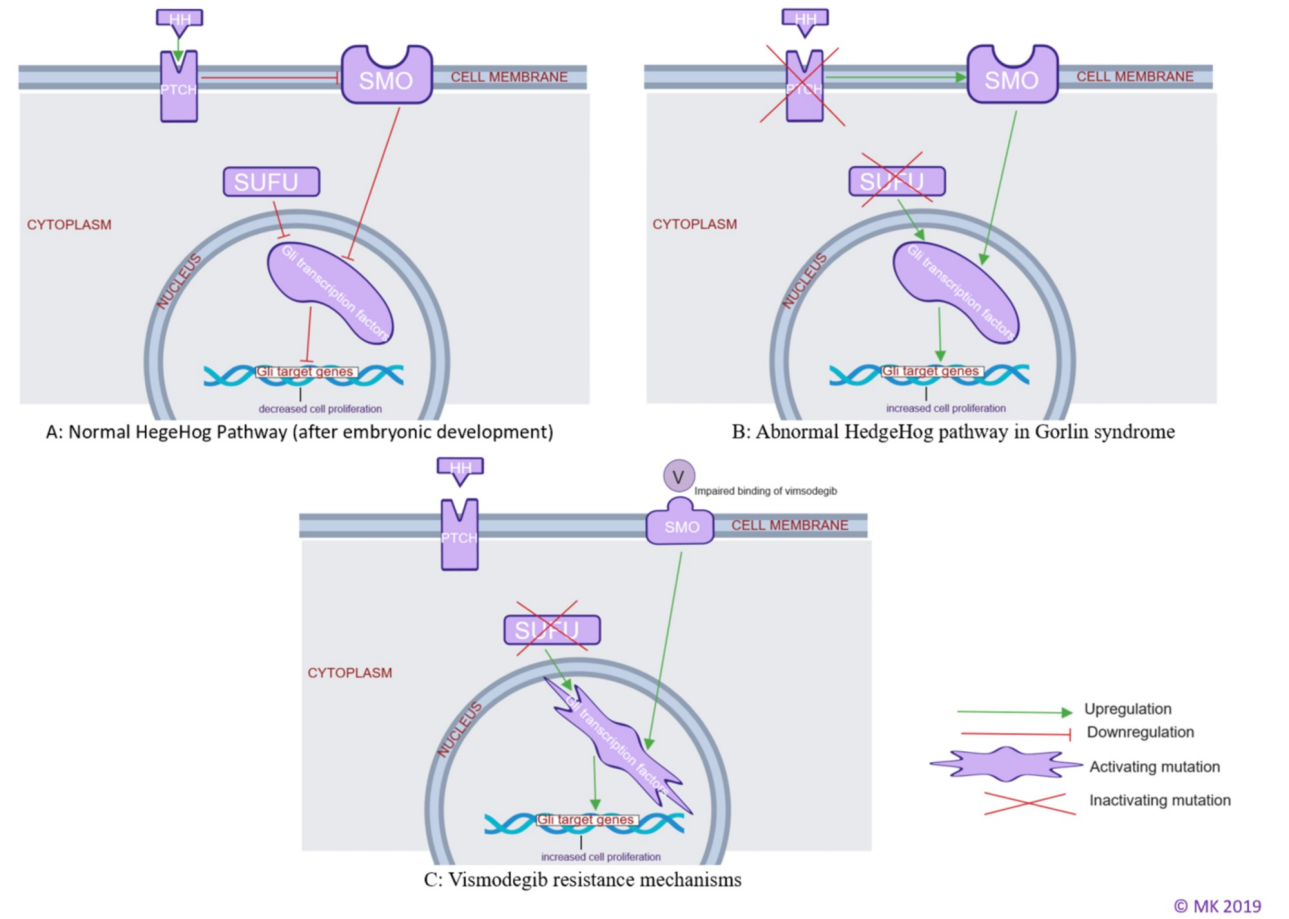
(A) Illustration showing normal hedgehog pathway, (B) illustration showing abnormal hedgehog pathway, (C) illustration showing vismodegib resistance.

The secreted hedgehog proteins act on the transmembrane receptor complex formed by two proteins-patched (PTCH) and smoothened (SMO). Smoothened (SMO) activation results in intracellular signal transduction leading to downstream activation of Gli nuclear transcription factors, which ultimately regulates hedgehog target gene expression causing cellular proliferation [8-9]. When hedgehog protein binds the transmembrane receptor complex, PTCH protein inhibits smoothened (SMO) through unclear mechanism resulting in downregulation of Gli transcriptions factors and keeps the cell proliferation under control [10]. The suppressor of fused (SUFU) proteins inhibit the Gli nuclear transcription factors directly, thereby keeping the cell proliferation under control as well (Figure 6A) [9,11-13].

Abnormal hedgehog pathway in NBCCS

Inactivating mutations of either patched (PTCH) or suppressor of fused (SUFU) genes lead to loss of inhibition of smoothened (SMO) and Gli nuclear transcription factors, respectively, leading to excess hedgehog (HH) target gene expression and thereby, excess cellular proliferation resulting in development of cancer (Figure 6B) [5,8].

Vismodegib resistance

In a study of 28 patients (including five patients with NBCCS), tumor regrowth during treatment with vismodegib occurred in six patients (21%) with a mean time to regrowth of 56 weeks [14-15]. The exact reason for this regrowth is unclear, and some theories attributing it to the development of acquired resistance to vismodegib have been proposed [16]. Tumor resistance likely develops from smoothened (SMO) protein mutation impairing its binding to vismodegib or due to mutations at the level of Gli transcription factors or SUFU proteins (which are downstream of SMO) [9,11-13]. It is unknown if these vismodegib-induced mutations in the smoothened (SMO) or the proteins downstream of SMO (like Gli or SUFU) lead to a more aggressive tumor/increased tumor burden compared to before vismodegib treatment (Figure 6C).

## Conclusions

Additional studies are needed to determine the optimal regimen, i.e., combining vismodegib with agents that inhibit smoothened (SMO) through different receptor or agents that inhibit hedgehog (HH) pathway downstream of smoothened (SMO). Additional studies are necessary to determine the optimal duration of vismodegib therapy, continuous treatment vs. discontinuation after best response, to prevent the development resistance to vismodegib. Further studies are required to determine if the development of resistance to vismodegib has any implications in the progression of Gorlin syndrome to a more aggressive version.
